# Borderline personality disorder and the big five: molecular genetic analyses indicate shared genetic architecture with neuroticism and openness

**DOI:** 10.1038/s41398-022-01912-2

**Published:** 2022-04-11

**Authors:** Fabian Streit, Stephanie H. Witt, Swapnil Awasthi, Jerome C. Foo, Martin Jungkunz, Josef Frank, Lucía Colodro-Conde, Guy Hindley, Olav B. Smeland, Tolou Maslahati, Cornelia E. Schwarze, Norbert Dahmen, Björn H. Schott, Nikolaus Kleindienst, Annette Hartmann, Ina Giegling, Lea Zillich, Lea Sirignano, Eric Poisel, Chi-Hua Chen, Markus M. Nöthen, Arian Mobascher, Dan Rujescu, Klaus Lieb, Stefan Roepke, Christian Schmahl, Martin Bohus, Stephan Ripke, Marcella Rietschel, Ole A. Andreassen

**Affiliations:** 1 Central Institute of Mental Health, Department of Genetic Epidemiology in Psychiatry, Medical Faculty Mannheim, Heidelberg University, Mannheim, Germany; 2 Central Institute of Mental Health, Center for Innovative Psychiatry and Psychotherapy, Medical Faculty Mannheim, Heidelberg University, Mannheim, Germany; 3 Department of Psychiatry and Psychotherapy, Charité Universitätsmedizin Berlin, Campus Mitte, Berlin, Germany; 4 Central Institute of Mental Health, Department of Psychosomatic Medicine and Psychotherapy, Medical Faculty Mannheim, Heidelberg University, Mannheim, Germany; 5 Section for Translational Medical Ethics, National Center for Tumor Diseases, German Cancer Research Center (DKFZ), Heidelberg, Germany; 6 QIMR Berghofer Medical Research Institute Brisbane, Brisbane, QLD Australia; 7 School of Psychology, University of Queensland, Brisbane, QLD Australia; 8 School of Biomedical Sciences, Queensland University of Technology, Brisbane, QLD Australia; 9 NORMENT, Centre for Mental Disorders Research, Division of Mental Health and Addiction, Oslo University Hospital, Oslo, Norway; 10 Psychosis Studies, Institute of Psychiatry, Psychology and Neurosciences, King’s College London, 16 De Crespigny Park, London, SE5 8AB United Kingdom; 11 Institute of Clinical Medicine, University of Oslo, Oslo, Norway; 12 Department of Psychiatry and Psychotherapy, Charité - Universitätsmedizin Berlin, Corporate Member of Freie Universität Berlin, Humboldt-Universität zu Berlin, Berlin Institute of Health, Campus Benjamin Franklin, Charité - Medical Faculty Berlin, Berlin, Germany; 13 Department of Developmental and Biological Psychology, Heidelberg University, Heidelberg, Germany; 14 Department of Psychiatry and Psychotherapy, University Medical Center, Mainz, Germany; 15 Leibniz Institute for Neurobiology, Magdeburg, Germany; 16 Department of Psychiatry and Psychotherapy, University Medical Center Göttingen, Göttingen, Germany; 17 Department of Psychiatry and Psychotherapy, Medical University of Vienna, Vienna, Austria; 18 Department of Neurology, Medical University of Vienna, Vienna, Austria; 19 Department of Radiology, University of California, San Diego, CA USA; 20 Institute of Human Genetics, University Hospital Bonn, Bonn, Germany; 21 Department of Psychiatry and Psychotherapy, St. Elisabeth Krankenhaus Lahnstein, Lahnstein, Germany; 22 Department of Clinical Psychology and Psychotherapy, Ruhr University Bochum, Bochum, Germany; 23 Broad Institute of MIT and Harvard, Stanley Center for Psychiatric Research and Medical and Population Genetics Program, Cambridge, MA USA; 24 Massachusetts General Hospital and Department of Medicine, Harvard Medical School, Analytic and Translational Genetics Unit, Boston, MA USA; 25 NORMENT Centre and KG Jebsen Centre for Neurodevelopmental disorders, Institute of Clinical Medicine, University of Oslo, Oslo, Norway; 26 Division of Mental Health and Addiction, Oslo University Hospital, Oslo, Norway

**Keywords:** Psychiatric disorders, Clinical genetics

## Abstract

Both environmental (e.g. interpersonal traumatization during childhood and adolescence) and genetic factors may contribute to the development of Borderline Personality Disorder (BPD). Twin studies assessing borderline personality symptoms/features in the general population indicate that genetic factors underlying these symptoms/features are shared in part with the personality traits of the Five Factor Model (FFM) of personality—the “Big Five”. In the present study, the genetic overlap of BPD with the Big Five -Openness to Experience, Conscientiousness, Extraversion, Agreeableness, and Neuroticism- was assessed. Linkage disequilibrium score regression was used to calculate genetic correlations between a genome-wide association study (GWAS) in central European populations on BPD (*N* = 2543) and GWAS on the Big Five (*N* = 76,551–122,886, Neuroticism *N* = 390,278). Polygenic scores (PGS) were calculated to test the association of the genetic disposition for the personality traits with BPD case-control status. Significant positive genetic correlations of BPD were found with Neuroticism (*rg* = 0.34, *p* = 6.3*10^−5^) and Openness (*rg* = 0.24, *p* = 0.036), but not with the other personality traits (all | *rg* | <0.14, all *p* > 0.30). A cluster and item-level analysis showed positive genetic correlations of BPD with the Neuroticism clusters “Depressed Affect” and “Worry”, and with a broad range of Neuroticism items (*N* = 348,219–376,352). PGS analyses confirmed the genetic correlations, and found an independent contribution of the personality traits to BPD risk. The observed associations indicate a partially shared genetic background of BPD and the personality traits Neuroticism and Openness. Larger GWAS of BPD and the “Big Five” are needed to further explore the role of personality traits in the etiology of BPD.

## Introduction

Borderline Personality Disorder (BPD) is a complex psychiatric disorder characterized by affective instability, identity disturbance, and interpersonal difficulties, and it is associated with high rates of self-injury and suicidal behaviors [1, 2]. It has been proposed that variants of normal personality traits contribute to the presentation of personality disorders [3–5]. This is also reflected in the introduction of the alternative DSM-5 model for personality disorders, i.e. that personality disorders are characterized by impairments in personality functioning and pathological personality traits and is also represented in the current concept of the ICD-11. The alternative DSM-5 model includes the domains of negative affectivity, detachment, psychoticism, antagonism, and disinhibition, which are variants of the five domains of the Big Five or the Five Factor Model (FFM) of personality (APA, 2013). The specific personality disorder diagnoses that can be derived from this model include antisocial, avoidant, borderline, narcissistic, obsessive-compulsive, and schizotypal personality disorders [1]. The Big Five -Openness to Experience (hereafter Openness), Conscientiousness, Extraversion, Agreeableness, and Neuroticism- each with six subdimensions or facets [6] can be measured with instruments like the Revised NEO Personality Inventory (NEO-PI-R) [7]. In the case of BPD, Lynam & Widiger [8] proposed a combination of high scores in specific Neuroticism and Openness facets and low scores in Agreeableness and Consciousness facets to distinguish those with BPD from others. This proposal has been confirmed in further studies [9].

Twin studies show that the expression of the “Big Five” personality traits is substantially influenced by genetic factors, with heritability estimates of 40–60% for different traits [10, 11]. Genetic factors also influence the risk for BPD: twin and family studies estimate the heritability to be around 46 [12] or 69% [13], indicating that besides well-established environmental risk factors such as early trauma or abuse [14, 15], the genetic background of an individual modulates their risk to develop BPD [16]. Moreover, twin studies indicate that the genetic factors underlying personality disorders and the Big Five personality traits are substantially shared [17–19] primarily indicating positive genetic correlations with Neuroticism, and negative genetic correlations with Agreeableness and Conscientiousness. More studies to investigate the overlap between the genetic factors influencing personality traits and those increasing the risk for personality disorders, including BPD, are needed [20, 21].

To further investigate the genetic overlap between personality traits and personality disorders observed in twin studies, data from genome-wide association studies (GWAS) can be used. GWAS systematically investigate the genetic underpinnings of a disorder or trait, by investigating the association of several million single nucleotide polymorphisms (SNPs)—common variations of one single nucleotide in the genetic code—with the phenotype of interest. The largest GWAS meta-analysis to date examining all Big Five personality traits (N = 76,551–122,886) [22], identified SNPs associated with Neuroticism, Consciousness, and Extraversion after rigid correction for multiple testing (*ɑ* = 5*10^−8^). More importantly this study by Lo et al. [22] showed a significant SNP-based heritability—the variance explained by the entirety of the investigated SNPs for all five personality traits (8.5–18%). A genetic principal component analysis showed the Big Five traits of Neuroticism and Openness to cluster with the genetics underlying several psychiatric disorders including affective disorders and Schizophrenia (SCZ) [22]. A larger GWAS meta-analysis by Nagel et al. [23] for Neuroticism including data from the UK Biobank (UKB) published shortly after (*N* = 449,484) [23] identified 136 independent associated genetic loci. Detailed analyses in the UK-Biobank subset (*N* = 348,219–376,352) where the 12-item Neuroticism scale of the EPQ-R [24] was applied, found evidence for substantial genetic heterogeneity within the scale of Neuroticism (in heritability and genetic association with other phenotypes) [25]. The authors identified two genetically distinguishable clusters of 4 items each, which were labeled “Depressed Affect” and “Worry”. Those clusters showed distinct genetic correlational patterns with other GWAS, notably also of mental disorders [23, 25], indicating that (genetic) analysis of personality traits should not be limited to the sum-score level.

Witt et al. [26] performed the first BPD case-control GWAS, comparing 998 patients with a diagnosis of BPD to 1545 controls. They did not observe associations on the level of single variants at the genome-wide significance level, but demonstrated that BPD has positive genetic correlations with Major Depressive Disorder (MDD), Bipolar Disorder (BD) and SCZ using linkage disequilibrium (LD) score regression. So far, molecular genetic approaches have not been used to investigate the association of BPD with the Big Five.

Our aim was to test whether the genetic variants associated with the risk of developing BPD are partially shared with the genetic variants associated with the Big Five personality dimensions. Therefore, we tested the genetic correlation of the BPD-GWAS by Witt et al. [26] with the Big Five GWAS by Lo et al. [22] except for Neuroticism, where we used the larger GWAS from Nagel et al. [25]. To explore the association of BPD with Neuroticism in a more detail, cluster and item-based genetic correlations were calculated for Neuroticism [23, 25]. As a complementary approach, polygenic scores (PGS) based on the Big Five GWAS were calculated in the Witt et al. [26] sample, and tested for their association with BPD case-control status.

## Methods

### Genetic correlations

We applied LD score regression [27], a method that incorporates information on the LD structure to estimate SNP-heritability and genetic correlations. LD score regression was carried out using a free intercept, and the 1000 Genomes data set served as a reference panel for underlying LD structure [28]. An overview of the used GWAS statistics can be found in Table 1.

**Table 1:** Overview of GWAS summary statistics analyzed.

Phenotype	Total N	Sample	Reference	See
Borderline Personality Disorder	2545	GBGC	26	Fig. 1, Fig. 2
Openness	76,581	23andMe and GPC	22	Fig. 1, Fig. 3
Conscientiousness	76,551			
Extraversion	122,886			
Agreeableness	76,551			
Neuroticism				
Sum score	390,278	UKB and GPC	23	Fig. 1, Fig. 3
Sum score UKB	380,506	UKB	25	Fig. 2
Clusters	348,219–357,957	UKB	23	Fig. 2
Single items	366,726–376,352	UKB	25	Fig. 2

*GBGC* German Borderline Genomics Consortium, *GPC* Genetics of Personality Consortium, *UKB* UK Biobank.

In a first step, the genetic correlations from GWAS results using the BPD-GWAS [26] (*N* = 2545) and the Big Five were calculated. For the Big Five the data from Lo et al. (*N* = 76,551–122,886) [22] was used, with the exception of Neuroticism, for which the larger meta-analysis by Nagel et al. was used (*N* = 390,278; excluding data from 23andMe Inc.) [25].

In a second step, to assess genetic correlations of BPD with Neuroticism clusters and items, we analyzed the genetic correlation of BPD [26] with the respective GWAS summary statistics from Nagel et al. based on the UKB (*N* = 348,219–376,352; UKB item codes 1920–2030) [23, 25].

Bonferroni corrected alpha levels were applied to the respective tests (BPD and BIG-5: *α* = 0.0033 (0.05/15 tested correlations; BPD and Neuroticism clusters/items *α* = 0.0033 (0.05/15 tested correlations).

### Polygenic Score analysis

#### Target sample

The BPD case-control GWAS has been described in detail previously [26]. Briefly, subjects fulfilling DSM-IV criteria for BPD and control subjects were recruited in clinical and research settings at three academic institutions in Germany (Mainz, Berlin, Mannheim). The study was approved by the local ethics committees, and all participants provided informed consent.

#### Polygenic score calculation

The PGS analysis was based on an updated quality control and imputation described in [29] carried out using the RICOPILI GWAS pipeline [30].

In brief, the sample was genotyped using the Infinium PsychArray-24 Bead Chip (Illumina, San Diego, CA, USA). Subjects and genetic markers were filtered using following criteria: individual and genotype missingness (≤2%), difference in missingness between in cases and controls (≤2%) and deviation from autosomal heterozygosity (|Fhet|>0.2) or from Hardy-Weinberg equilibrium (*p* < 1*10^−6^ in controls; p < 1*10^−10^ cases). Additionally, genetic outliers, sex-mismatches and cryptic related subjects (pi-hat>0.2) were removed. After quality control the sample comprised 998 cases and 1545 controls.

Imputation was carried out using a publicly available reference panel from the haplotype reference consortium (EGAD00001002729) with EAGLE/MINIMAC3 (default parameters; variable chunk size of 132 genomic chunks) [31, 32].

PGS for the five Big Five traits were generated for each individual in the target sample using the Big Five GWAS as discovery samples with PRSice2 [33]. PGS were calculated by summing the allele counts of the respective SNPs weighted by the GWAS effect sizes using standard settings and excluding the extended major histocompatibility complex region (ch6 26–33MB) because of its extended LD structure. PGS were calculated for the following p-value thresholds (*PT*: 5*10^−8^, 1*10^−6^, 1*10^−4^, 0.001, 0.01, 0.05, 0.1, 0.2, 0.5 and 1.0).

### Statistical analysis

The association of the generated PGS with BPD case-control status was assessed in PRSice2 [33] using logistic regression models with case-control status as the dependent variable. *Nagelkerke-pseudo-R²* (*NkR²*) was calculated as effect measure, corresponding to *R²* increase when adding the PGS to a model only containing the covariates (first 10 ancestry components PC1-PC10). We report uncorrected two sided significance levels, and adjusted alpha levels corresponding to a Bonferroni correction for the 10 tested p-value thresholds per PGS (α = 0.005; 0.05/10 tested associations).

In a second step, to test the independent genetic contribution of different Big Five traits to BPD risk, we selected the PGS showing the most significant association with case-control status for each Big Five factor (in case *p* was < 0.005) in a joint logistic regression model with case-control status as dependent variable and the selected PGSs and the first 10 PCs as covariates. Analyses were conducted in the R statistical environment (v 3.5.1).

## Results

### Genetic correlations

**Fig. 1:**
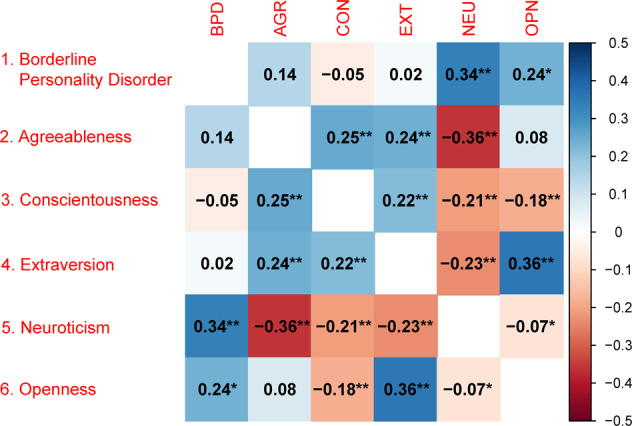
Genetic correlations between Borderline Personality Disorder and the Big Five personality traits. *Note:* Red fields indicate negative and blue fields indicate positive genetic correlations. * *p* < 0.05, ** *p* < 0.0033 (0.05/15 tested correlations). Neuroticism was based on Nagel et al. [23], the other BIG-5 personality traits were based on Lo et al. [22].

As shown in Fig. 1, significant positive genetic correlations were observed between Agreeableness, Conscientiousness, Extraversion and Openness, with the exception of a negative correlation of Conscientiousness with Openness. Neuroticism was negatively correlated with the other personality traits of the Big Five. The correlational patterns correspond largely to those reported in Lo et al. [22], even when using the larger Neuroticism GWAS meta-analysis by Nagel et al. [23, 25].

For BPD, the LD-score regression SNP-heritability estimate was 50.3% (95% CI = 17,9–85,3%) on the liability scale for a population prevalence of 3% [34, 35]. BPD showed a statistically significant positive genetic correlation with Neuroticism (rg = 0.34, *p* = 6.3*10^−5^) and nominally significant correlation with Openness (rg = 0.24, *p* = 0.036) (see Fig. 1 and Table 2). No significant genetic correlations were observed with the other Big Five traits.

**Table 2:** Genetic correlations of Borderline Personality Disorder with Big Five personality traits.

Big Five Trait	*rg*	*se*	*z*	*p*
**Agreeableness**	0.14	0.14	1.03	0.30
**Conscientiousness**	−0.05	0.11	−0.41	0.68
**Extraversion**	0.02	0.10	0.22	0.83
**Neuroticism**^**#**^	0.34**	0.08	4.00	6.3*10^−5^
**Openness**	0.24*	0.11	2.09	0.036

*Note:* * *p* < 0.05, ** *p* < 0.0033 (0.05/15 tested correlations), ^#^based on Nagel et al. [23], the other BIG-5 personality traits were based on Lo et al. [22].

**Fig. 2:**
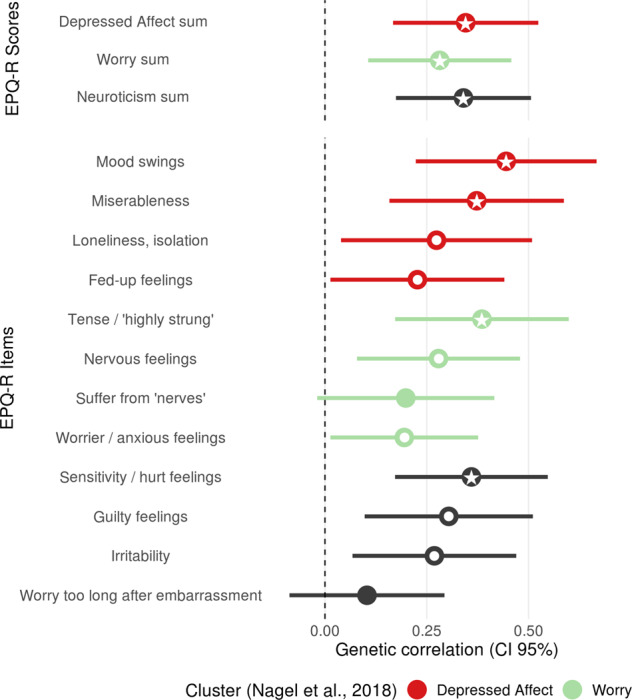
Genetic correlations between Borderline Personality Disorder with clusters and single items of the EPQ-R Neuroticism scale. *Notes*. Colors indicate membership to genetic clusters: red = “Depressed Affect”, green = “Worry”, dark grey = not assigned to a cluster. All data is based on the UK Biobank data sample [23, 25]. Within each cluster category, items are sorted by their genetic correlation with BPD; • *p* < 0.05, * *p* < 0.0033 (0.05/15 tested correlations). 95% CI 95% confidence interval, EPQ-R Eysenck Personality Questionnaire Revised.

Both the “Depressed Affect” cluster (*rg* = 0.35, *p* = 1.5*10^−4^), and the “Worry” cluster (*rg* = 0.28, *p* = 1.7*10^−3^) were significantly associated with BPD. On a single item level, some degree of heterogeneity of the genetic correlation of BPD with Neuroticism was observed. While all observed genetic correlations were positive, the correlations ranged from 0.10 to 0.45. Descriptively, with items from the “Depressed Affect” cluster, BPD showed the strongest associations with the items “mood swings” and “miserableness”. From the “Worry” cluster, only the correlation with “tense/‘highly strung’” was significant after correction for multiple testing. From the items not assigned to either of the clusters, “sensitivity/hurt feelings” showed descriptively the strongest correlation with BPD (see Fig. 2).

### Polygenic score analysis

**Fig. 3:**
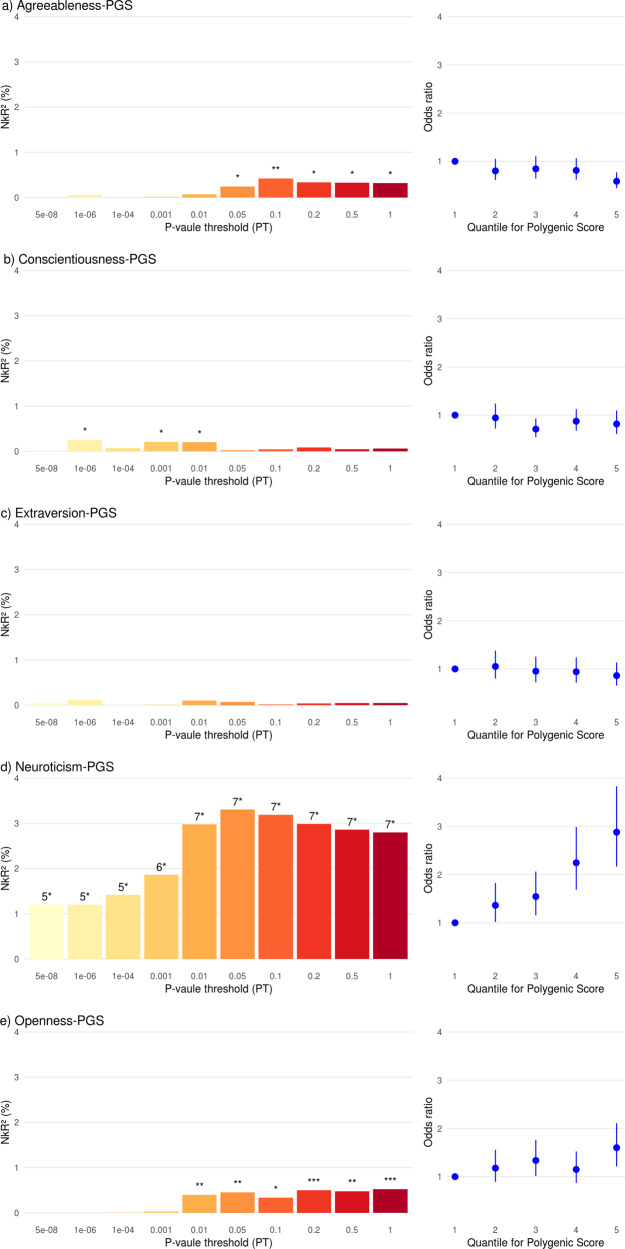
Association of Big Five Polygenic Scores (PGS) with Borderline Personality Disorder case-control status. Left panels: Proportion of explained variance in case-control status (*Nagelkerke R²*) depicted for the ten tested *p* value thresholds (PT). Right panels: Odds Ratio for BPD by PGS quintile, with decile 1 as reference. Regressions were computed with the first 10 ancestry principle components to adjust for population stratification. * *p* < 0.05; ** *p* < 0.005 (0.05/10 tested *p*-value thresholds per PGS); *** *p* < 0.001 (0.05/10 tested *p*-value thresholds /5 personality traits); 4* *p* < 1*10^−4^; 5* *p* < 1*10^−5^; 6* *p* < 1*10^−8^; 7* *p* < 1*10^−12^. Neuroticism-PGS was based on Nagel et al. [23], the other BIG-5 personality traits PGS were based on Lo et al. [22].

PGS of Openness (strongest association at *PT* = 1; *NkR*^2^ = 0.52%, *p* = 0.00073) and Neuroticism (*PT* = 0.05; *NkR*^2^ = 3.3%, *p* = 4.5*10^−17^) showed a positive association with BPD case-control status, corresponding to higher PGSs in the cases. Agreeableness showed a negative association with case-control status at one threshold (*PT* = 0.05; *NkR*^2^ = 0.42%, *p* = 0.0024), and no significant associations were observed for the PGS for Conscientiousness and Extraversion (see Fig. 3 and Supplementary Tables 1-5).

A joint regression model including the significant PGS, explained 4.4% of the variance (*NkR*^*2*^) in BPD case–control status, and indicated an independent contribution of Neuroticism (*p* = 2.2*10^−16^), Openness (*p* = 0.00032) and Agreeableness (*p* = 0.0060).

## Discussion

The present study is the first to use molecular genetic data to investigate shared genetic factors between diagnosed BPD and the Big Five personality traits, showing a genetic correlation of BPD with the trait Neuroticism and a suggestive correlation with Openness. The genetic correlations were confirmed by PGS analyses, which found increased PGS of the two Big Five traits in BPD cases compared to controls, with the joint analysis indicating an independent contribution to BPD risk. These results provide biological evidence that partially supports the concept that associates variants of normal personality traits and the presence of BPD. Specifically, it supports the description of BPD as high levels of neuroticism and openness, although the original proposal includes also low levels of agreeableness and conscientiousness [8]. Notably, while we did not observe a negative genetic correlation between agreeableness and BPD, we observed lower Agreeableness-PGS in BPD cases compared to controls.

The observed genetic association of BPD and Neuroticism is in line with findings from twin and family studies, showing a positive genetic overlap of Neuroticism and Borderline Personality Features [19], a continuous measure of borderline personality assessed with the Personality Assessment Inventory–Borderline Features scale (PAI-BOR) which can be used in the general population [36] or BPD DSM-IV Criterion counts [17]. A polygenic risk score summarizing the genetic risk burden for Borderline Personality Features [37] was associated with Neuroticism (smallest *p* = 5.43*10^−7^) in a target sample of >100,000 subjects from the general population [38], supporting a genetic relation between both phenotypes.

Phenotypic studies implicate that Neuroticism increases risk for most psychiatric disorders [39–41]. It is likely that a genetic disposition to Neuroticism also contributes to genetic correlations between many psychiatric disorders [42] and the observed comorbidities [43]. With respect to depression, strong genetic correlations of Neuroticism with both depressive symptoms in the general population as well as with clinical MDD have been observed [22, 44]. As a broad genetic risk factor, Neuroticism might link BPD to other (comorbid) psychiatric conditions as well as other personality disorders [45]. Supporting this, a twin study showed that the finding of a genetic association between Borderline Personality Features and substance use disorders, is attributable to variation in personality factors, especially Neuroticism [46].

Neuroticism comprises facets which are characteristic clinical features of BPD personality, such as emotional lability, anxiousness, angry hostility, depressiveness, and vulnerability e.g. [4]. In the present study, BPD showed similar correlations with both tested Neuroticism clusters, with slightly a higher correlation for “Depressed Affect”. In the item-based analysis, all 12 items showed a positive correlation with BPD, with nominal significance for 10, and significance after Bonferroni correction for four of the 12 items. The results indicate that a rather broad range of aspects of Neuroticism contribute to BPD, similar to depression and anxiety, and distinct from disorders such as schizophrenia, anorexia nervosa or attention deficit hyperactivity disorder, which show primarily association with one of the two clusters [25]. Notably, the descriptively most strongly correlated item was “mood swings” which has previously been used as a measure of mood instability [47], a core symptom of BPD [48]. “Mood swings” has shown extensive genetic overlap with a range of mental disorders, with the strongest positive correlations being reported for depression, anxiety, ADHD and PTSD [47, 49]. Future research should further investigate how single items, and specific clusters of these features contribute to different psychiatric disorders [50].

Less is known about the genetic correlation of Openness with BPD and other psychiatric disorders, although high level of Openness to feelings and to actions have been proposed to characterize BPD [8]. In twin studies, Openness for Experience showed a moderate genetic correlation with DSM-IV Criterion counts for BPD (*rg* = 0.24) [17] but no evidence for a genetic correlation with Borderline Personality Features [19]. Using a molecular genetic approach, we observed a nominally significant genetic correlation (*rg* = 0.24), and -with stronger significance- higher Openness-PGS in BPD patients compared to controls. These disparities may be attributable to the different instruments used in the studies, or to the fact that the present results are based on a sample of patients fulfilling the diagnosis of BPD [26], and not on normal variation observed in Borderline Personality Features in unaffected subjects. It is unclear, however, if this might entail differences in statistical power, or in the underlying genetic architecture.

While Openness is generally considered a beneficial personality trait, in high levels and in combination with other traits it might be disadvantageous and increase the risk for certain psychiatric disorders [51]. The present results suggest that increased Openness and BPD might be influenced by overlapping genetic factors. Openness has for example been associated with aspects of risk-taking including substance use [52, 53], and increased risk-taking is a characteristic feature of BPD, which contributes strongly to the impairment experienced by affected patients and their relatives. Furthermore, both Openness [54] and BPD [55] are associated with hallucinations; shared genetic variants associated might underlie these associations. Openness has been shown to be genetically associated to other psychiatric disorders such as BD and SCZ [22], and BPD and SCZ share a sizable fraction of genetic risk factors [26]. Still, compared to Neuroticism, Openness has been less strongly linked with BPD, and while all facets of Neuroticism are related to the BPD phenotype, only Openness to feelings and actions do [51]. In regard to the association of BPD with other Big Five traits, there are inconsistencies with previous studies: the results of twin studies [17, 19] and clinical observations [51] indicate a negative association of Borderline Personality with Agreeableness and Conscientiousness, whereas we observe genetic correlations close to zero. However, the observed lower PGS for Agreeableness in the BPD patients, is in line with the twin and clinical studies.

There are some limitations to the study: First, while BPD showed genetic correlations with two of the Big Five, the effects and the statistical significance of the results are limited in the case of Openness, where the association did not survive correction for multiple testing. Larger samples are needed to confirm and expand the observed associations: despite being the largest available samples for the phenotypes, the samples for the Big Five and especially, the BPD GWAS are limited in size. Indeed, the z-score of the SNP-heritability of BPD is lower than the threshold of 4, which is recommended for robust genetic correlation analysis with LDSC. However, the PGS analyses confirm the association of the two personality traits with BPD, with significant results being observed for both traits after correction for multiple testing. The observed negative association of Agreeableness PGS with BPD might reflect the higher sensitivity of the PGS method in a sample of the present size; however, this result needs to be replicated in future analyses. Increased sample sizes will be able to provide more reliable estimates of the underlying genetic risk variants. The sample of BPD is drastically smaller compared to those of other psychiatric disorders such as MDD [56], BD [57], and SCZ [58]. Clinicians and researchers in the field of BPD should work together to facilitate the generation of larger BPD case-control samples for genetic studies. Second, we assessed genetic correlations to assess shared genetic overlap. However, more complex genetic overlap, with a mix of agonistic and antagonistic shared effects can remain undetected by this approach, as it has been shown e.g. for bipolar disorder and cognition [59]. Third, the participation of subjects with a history of BPD and marked personality trait profiles in the Big Five GWAS might have influenced our results. However, given the prevalence of BPD, it is unlikely that this contributed to a large degree to our results. However, we cannot rule out, that the presence of more common psychiatric conditions, such as depression, which is associated with Neuroticism, and often comorbid to BPD might have influenced the results. Fourth, different personality inventories were used in the different studies to assess the personality traits. However, high genetic correlations were reported for the personality traits between the samples (all rg > .83) [22, 23]. Fifth, the available samples were all from European ancestry, which limits the transferability of the results to other populations. Furthermore, the GWAS by Lo et al. [22] investigated sum scores of personality domains, but did not investigate the respective facets. While our cluster and item-based analyses for Neuroticism indicate specific contributions of personality clusters/facets, no item-based data was available to us for the other Big Five traits. Future genetic studies of BPD and the Big Five and should assess the Big Five and their facets in more detail.

The present study shows the applicability of molecular genetic approaches to investigating the extent to which common genetic factors underlie personality traits and BPD. Future research should extend the present analysis, by leveraging data from large and well characterized samples. Besides detailed analyses of the underlying personality facets, future research should investigate functional domains involved in BPD and the Big Five, as formulated in the Research Domain Criteria (RDoC) approach [60]. The present study investigated genetic associations of BPD with the Big Five. It should be noted that environmental factors such as early or recent trauma and chronic stress, which are highly important contributors to BPD [14, 15, 61], influence the development of personality not only in early age, but also throughout the entire lifetime [62]. Identifying and assessing the relevant aspects of environmental exposure suspected to modify biological pathways is a major challenge, especially when the pathways are thought to link normal variation in personality traits to personality disorders. Future studies integrating environmental aspects in as much detail and as reliably as possible will be able to further address the interplay of environmental and genetic factors in the analyses (e.g. [63, 64]). While the present study indicates the polygenic nature of the genetic contribution to BPD risk, and a genetic overlap between specific personality factors and BPD risk, it is also apparent from the PGS analyses that the predictive power of genetic studies for BPD risk is still very low, and not yet of clinical or diagnostic utility. In this context, molecular genetic studies investigating the potential of genetic measures to discriminate between different disorders, or to predict disorder course or treatment response are of interest for future studies.

In summary, the present study gives the first molecular genetic insight into the shared genetic factors underlying BPD and normal variation in the Big Five personality traits and supports the relationship of BPD with Openness and Neuroticism. Future studies may extend this approach to the specific underlying genetic variants and other psychiatric conditions, thereby helping to further elucidate the relationship between psychopathology and normal variability of human personality.

## Supplementary information

Supplementary Tables

## Data Availability

All GWAS summary statistics used for Neuroticism sum scores, items or clusters [23, 25] are publicly available (https://ctg.cncr.nl/software/summary_statistics). GWAS summary statistics of the other Big Five [22], include data from 23andme and can be made available to qualified investigators who enter into an agreement with 23andMe that protects participant confidentiality.

## Code availability

The BPD summary statistics and analysis code are available from the corresponding author on reasonable request.
