# Supplementary material for: Borderline personality disorder and the big five: molecular genetic analyses indicate shared genetic architecture with neuroticism and openness
Source: Transl Psychiatry. 2022 Apr 11;12:153. doi: 10.1038/s41398-022-01912-2 (PMC9001677; doi:10.1038/s41398-022-01912-2)
Supplement: Supplementary file 1 — Supplementary Tables [file 41398_2022_1912_MOESM1_ESM.docx]

**Supplementary Tables**

**Streit et al. - Borderline Personality Disorder and the Big Five: Molecular Genetic Analyses Indicate Shared Genetic Architecture with Neuroticism and Openness**

Table S1: Prediction of Borderline Personality Disorder case-control status with Agreeableness PGS

| Threshold | NkR2 | P | Coefficient | Standard.Error | Num_SNP |
| --- | --- | --- | --- | --- | --- |
| 1.0E-06 | 0.06% | 0.2673 | 0.0566 | 0.0510 | 3 |
| 0.0001 | 0.0004% | 0.9299 | -0.0455 | 0.5173 | 120 |
| 0.001 | 0.01% | 0.5694 | -0.9804 | 1.7231 | 1104 |
| 0.01 | 0.07% | 0.2293 | -7.2020 | 5.9903 | 8822 |
| 0.05 | 0.24% | 0.0213 | -33.6747 | 14.6299 | 34729 |
| 0.1 | 0.42% | 0.0024 | -66.2673 | 21.8206 | 61876 |
| 0.2 | 0.33% | 0.0070 | -89.5139 | 33.1971 | 107871 |
| 0.5 | 0.32% | 0.0078 | -157.3020 | 59.0859 | 213693 |
| 1 | 0.32% | 0.0084 | -224.886 | 85.3082 | 312837 |

Table S2: Prediction of Borderline Personality Disorder case-control status with Conscientiousness PGS

| Threshold | NkR2 | P | Coefficient | Standard.Error | Num_SNP |
| --- | --- | --- | --- | --- | --- |
| 5.0E-08 | 0.01% | 0.6716 | -0.0128 | 0.0302 | 1 |
| 1.0E-06 | 0.26% | 0.0174 | -0.1318 | 0.0554 | 4 |
| 0.0001 | 0.07% | 0.2191 | -0.6406 | 0.5213 | 152 |
| 0.001 | 0.21% | 0.0312 | -3.8906 | 1.8061 | 1190 |
| 0.01 | 0.20% | 0.0356 | -12.8254 | 6.1043 | 8687 |
| 0.05 | 0.02% | 0.5176 | -9.5155 | 14.7049 | 34252 |
| 0.1 | 0.05% | 0.3021 | -22.6868 | 21.9861 | 61066 |
| 0.2 | 0.08% | 0.1799 | -44.9355 | 33.5093 | 107225 |
| 0.5 | 0.05% | 0.3087 | -60.6047 | 59.5350 | 213292 |
| 1.0 | 0.06% | 0.2605 | -96.7189 | 85.9617 | 312679 |

Table S3: Prediction of Borderline Personality Disorder case-control status with Extraversion PGS

| Threshold | NkR2 | P | Coefficient | Standard.Error | Num_SNP |
| --- | --- | --- | --- | --- | --- |
| 5.0E-08 | 0.05% | 0.2950 | -0.0541 | 0.0517 | 5 |
| 1.0E-06 | 0.11% | 0.1119 | -0.1078 | 0.0678 | 11 |
| 0.0001 | 0.0005% | 0.9183 | 0.0536 | 0.5224 | 239 |
| 0.001 | 0.01% | 0.6364 | 0.7824 | 1.6548 | 1408 |
| 0.01 | 0.10% | 0.1394 | 8.1939 | 5.5438 | 8994 |
| 0.05 | 0.07% | 0.2300 | 17.0519 | 14.2041 | 35185 |
| 0.1 | 0.01% | 0.5884 | 11.6527 | 21.5321 | 62495 |
| 0.2 | 0.03% | 0.3816 | 28.8323 | 32.9521 | 108653 |
| 0.5 | 0.05% | 0.2979 | 61.3750 | 58.9562 | 213295 |
| 1.0 | 0.05% | 0.3180 | 85.2988 | 85.4219 | 312494 |

Table S4: Prediction of Borderline Personality Disorder case-control status with Neuroticism PGS

| Threshold | R2 | P | Coefficient | Standard.Error | Num_SNP |
| --- | --- | --- | --- | --- | --- |
| 5.0E-08 | 1.20% | 2.99E-07 | 1.0023 | 0.1956 | 131 |
| 1.0E-06 | 1.20% | 3.00E-07 | 1.7362 | 0.3389 | 287 |
| 0.0001 | 1.42% | 2.88E-08 | 5.8687 | 1.0577 | 1436 |
| 0.001 | 1.86% | 2.26E-10 | 15.9010 | 2.5070 | 4580 |
| 0.01 | 2.98% | 1.39E-15 | 56.7002 | 7.0999 | 18296 |
| 0.05 | 3.31% | 4.46E-17 | 136.2500 | 16.2201 | 54232 |
| 0.1 | 3.19% | 1.43E-16 | 193.7840 | 23.4538 | 87991 |
| 0.2 | 2.99% | 1.27E-15 | 279.1380 | 34.9043 | 142750 |
| 0.5 | 2.86% | 5.17E-15 | 465.0530 | 59.4493 | 261476 |
| 1.0 | 2.80% | 9.51E-15 | 642.1560 | 82.9060 | 368944 |

Table S5: Prediction of Borderline Personality Disorder case-control status with Openness PGS

| Threshold | NkR2 | P | Coefficient | Standard.Error | Num_SNP |
| --- | --- | --- | --- | --- | --- |
| 1.0E-06 | 0.0001% | 0.9611 | -0.0040 | 0.0823 | 8 |
| 0.0001 | 0.003% | 0.8039 | -0.1282 | 0.5164 | 161 |
| 0.001 | 0.03% | 0.4086 | 1.4044 | 1.6995 | 1174 |
| 0.01 | 0.40% | 0.0032 | 17.9506 | 6.0843 | 8912 |
| 0.05 | 0.45% | 0.0016 | 47.1867 | 14.9432 | 35004 |
| 0.1 | 0.33% | 0.0069 | 59.1348 | 21.8727 | 62107 |
| 0.2 | 0.50% | 0.0010 | 110.7920 | 33.5503 | 108561 |
| 0.5 | 0.48% | 0.0012 | 192.5660 | 59.4907 | 213675 |
| 1 | 0.52% | 0.0007 | 291.456 | 86.3208 | 312690 |
